# Supplementary material for: FOXA2 promotes metastatic competence in small cell lung cancer
Source: Nat Commun. 2025 May 26;16:4865. doi: 10.1038/s41467-025-60141-5 (PMC12106783; doi:10.1038/s41467-025-60141-5)
Supplement: Supplementary file 1 — Supplementary Information [file 41467_2025_60141_MOESM1_ESM.docx]

**Supplementary Information**

**FOXA2 promotes metastatic competence in small cell lung cancer**

Kenta Kawasaki^1,2^, Sohrab Salehi^3^, Yingqian A. Zhan^4^, Kevin Chen^5^, Jun Ho Lee^1^, Eralda Salataj^4^, Hong Zhong^2^, Parvathy Manoj^2^, Dennis Kinyua^2^, Barbara P. Mello^2^, Harsha Sridhar^2^, Sam E. Tischfield^6^, Irina Linkov^7^, Nicholas Ceglia^3^, Matthew Zatzman^3^, Eliyahu Havasov^3^, Neil J. Shah^2^, Fanli Meng^6^, Brian Loomis^6^, Umesh K. Bhanot^7^, Esther Redin^2^, Elisa de Stanchina^5^, Pierre-Jacques Hamard^4^, Richard P. Koche^4^, Andrew McPherson^3^, Álvaro Quintanal-Villalonga^2^, Sohrab P. Shah^3^, Joan Massagué^1,8,^*, Charles M. Rudin^2,8,^*

^1^Cancer Biology and Genetics Program, Sloan Kettering Institute, Memorial Sloan Kettering Cancer Center, New York, NY 10065, USA

^2^Department of Medicine, Memorial Sloan Kettering Cancer Center, New York, NY, USA

^3^Computational Oncology Program, Memorial Sloan Kettering Cancer Center, New York NY, USA

^4^Center for Epigenetics Research, Memorial Sloan Kettering Cancer Center, New York, NY, USA

^5^Antitumor Assessment Core, Memorial Sloan Kettering Cancer Center, New York, NY, USA

^6^ Marie-Josée and Henry R. Kravis Center for Molecular Oncology, Memorial Sloan Kettering Cancer Center, New York, NY, USA

^7^ Pathology Core Facility, Memorial Sloan Kettering Cancer Center, New York, NY, USA.

^8^Weill Cornell Medicine Graduate School of Medical Sciences, New York, NY, USA

**Supplementary figures and figure legends:**

**
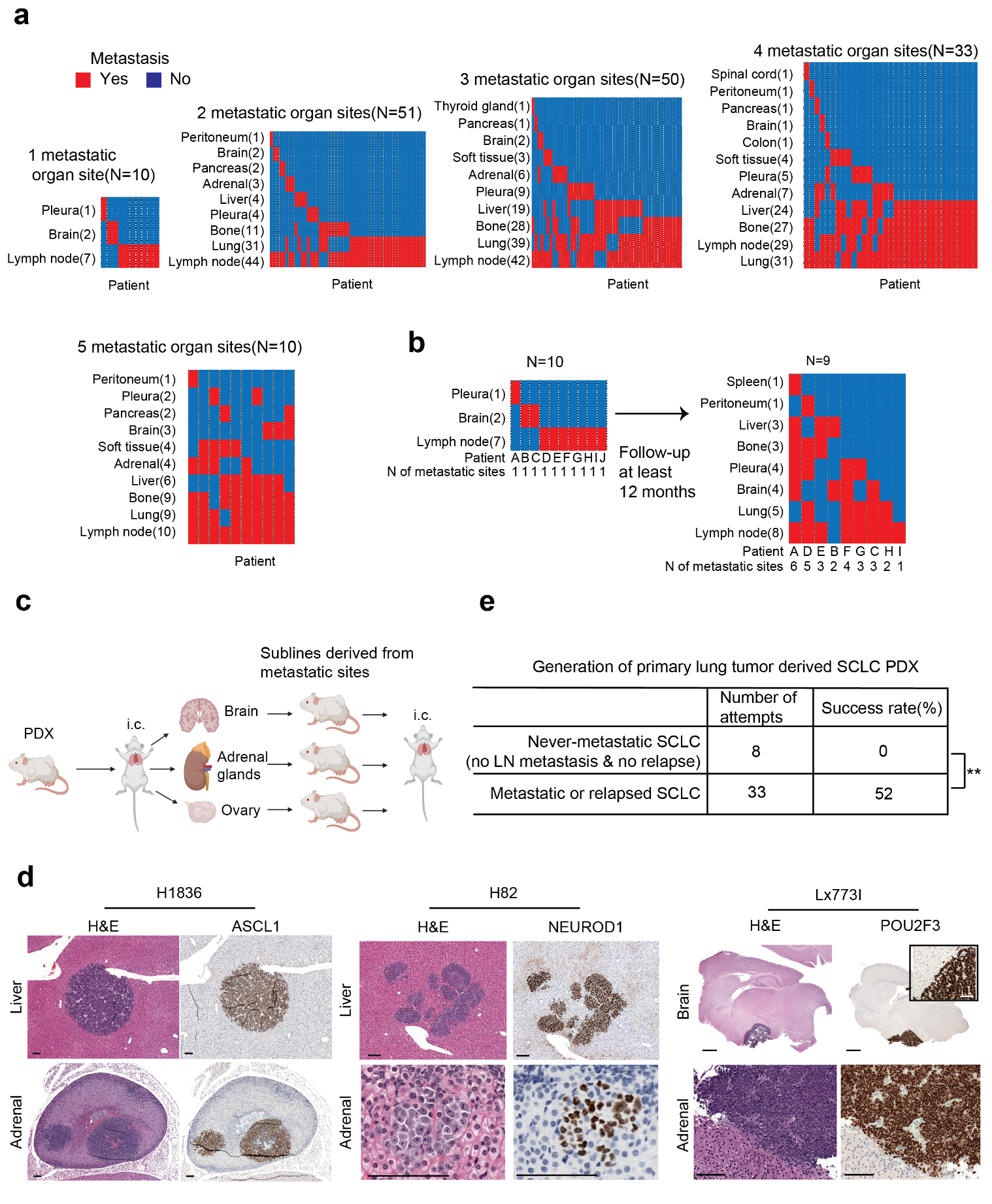
**

**Supplementary Figure 1 related to Figure 1**

**a:** Distribution of SCLC metastases (red squares) to organ sites. Blue squares indicate no metastases to each organ in each patient. Metastatic sites are shown and the number in the bracket indicates the number of the patients. **b:** Follow-up (12-month minimum) of SCLC patients who had only 1 metastatic site at baseline. **c:** Experimental design schematic for metastatic subline generation from SCLC PDX. Created in BioRender. Rudin C. (2025) <https://BioRender.com/jb4h810>. i.c.: intra-cardiac injection. **d:** H&E and IHC staining (ASCL1, NEUROD1, POU2F3) of each cell line and PDX line used for the metastasis assay. Scale bar:1000μm (Lx773I brain, inset is 100μm) and 100μm. **e:** Success rate of PDX generation of primary SCLC lung tumors. Never-metastatic SCLC include tumors without lymph node metastasis at diagnosis, and no subsequent relapse. Metastasized or relapsed SCLC include tumors that had metastasized at diagnosis, or that relapsed after treatment (** < 0.01, *P*=7.84x10^-3^, Chi-squared test).

**
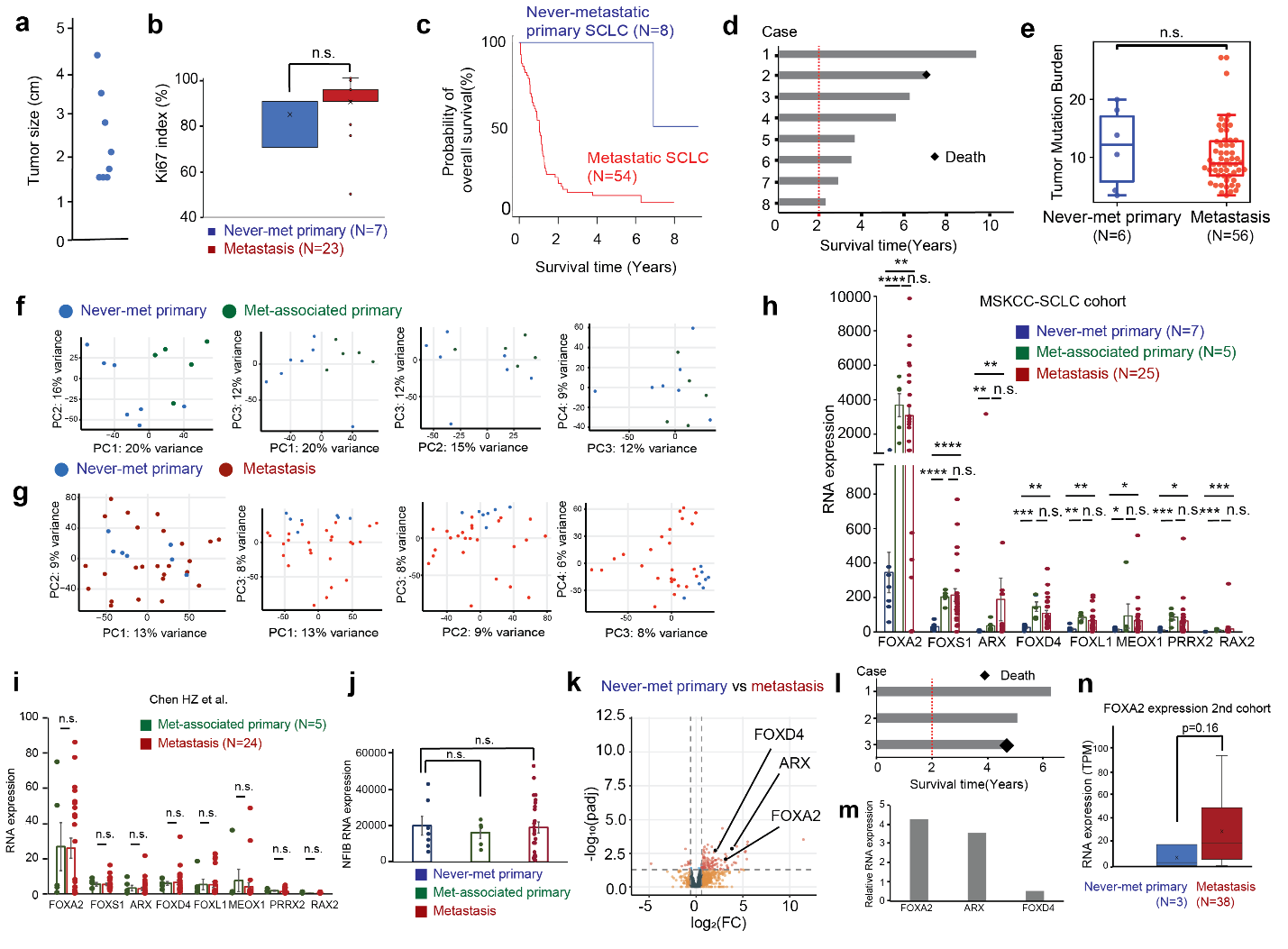
**

**Supplementary Figure 2 related to Figure 2**

**a:** Distribution of tumor size in never-metastatic primary SCLC(N=8). **b:** Comparison of Ki67 index in never-met primary and metastasis group. **c:** Overall survival data of patients with never-metastatic vs. metastatic SCLC. **d:** Swimmers plot of survival of patients with never-met primary tumor (N=8). **e:** Comparison of tumor mutation burden in never-met primary and metastasis group. **f, g:** PCA of gene expression in never-met primary SCLC vs met-associated primary SCLC (**f**) and never-met primary SCLC vs metastatic SCLC (**g**). **h:** Bar graph for relative expression of the 8 nominated TF genes in never-met primary SCLC, met-associated primary SCLC, and SCLC metastases. Graphs indicate mean±SEM (* < 0.05, ** < 0.01, *** < 0.001, **** < 0.0001, from left to right; FOXA2; *P*=7.31x10^-6^*, P*=8.81x10^-3^,FOXS1; *P*=1.09x10^-5^*, P*=4.47x10^-5^, ARX; *P*=7.56x10^-3^, *P*=1.38x10^-3^, FOXD4; *P*=4.18 x10^-4^, *P*=1.92x10^-3^, FOXL1; *P*=1.32x10^-3^, *P*=9.88x10^-3^, MEOX1; *P*=0.0311, *P*=0.0311, PRRX2; *P*=3.58x10^-4^*, P*=0.0137, RAX2; *P*=6.32x10^-4^*, P*=9.48x10^-4^, Wald’s t-test). **i:** Bar graph of expression of the 8 nominated TF genes in met-associated primary lung and metastases in publicly available SCLC autopsy RNA seq data^3^. Graphs indicate mean±SEM (two-sided Student’s t-test). **j:** RNA expression of NFIB in the three groups indicated. Mean±SEM (one-way ANOVA). **k:** Volcano plot of candidate genes in bulk RNA-seq data (never-met primary SCLC vs metastasis). **l:** Swimmers plot of survival duration in never-met primary SCLC cases in a 2^nd^ independent MSKCC cohort. **m:** Relative expression of the 3 selected TFs in the 2^nd^ MSKCC cohort. **n:** *FOXA2* expression in the 2^nd^ MSKCC cohort. Mean±SEM (two-sided Student’s t-test).


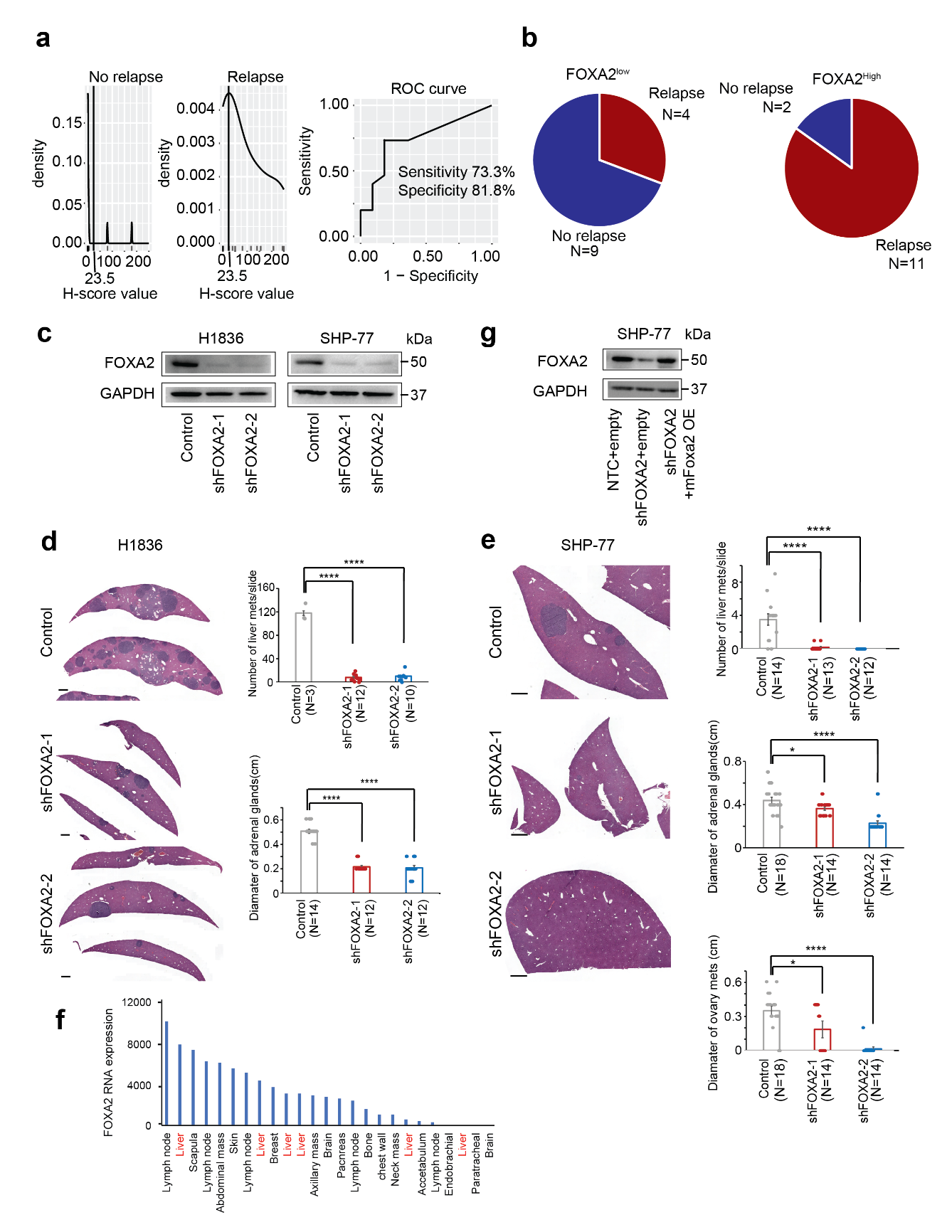


**Supplementary Figure 3 related to Figure 3**

**a:** FOXA2 H-scores, determination of an optimal cut point to distinguish by class (no relapse versus relapse) for outcome among patients with limited stage SCLC. Receiver operating characteristic (ROC) curve demonstrates sensitivity 73.3% and specificity 81.8%. **b:** Distribution of patients with and without relapse in FOXA2^low^ vs. FOXA2^high^ cohorts. **c:** Western blot of FOXA2 KD in H1836, SHP-77 cell lines. The samples derive from the same experiment but different gels for FOXA2, another for GAPDH were processed in parallel. A representative data of one of the 3 individual experiments. **d, e:** Gross evaluation of liver, adrenal gland, and ovary metastases in FOXA2 KD vs. controls for cell lines H1836 and SHP-77. Scale bar: 500μm. **f**: FOXA2 expression in metastasis samples. **g:** Western blot of FOXA2 restoration in SHP-77 FOXA2 KD by mouse Foxa2 (mFoxa2). A representative data of one of the 3 individual experiments. Bar graphs display mean±SEM by cohort (* < 0.05, **** < 0.0001, from left to right; **d** *P*=1.01x10^-11^*, P*=1.32x10^-9^ (top), *P*=5.69x10^-10^*, P*=5.68x10^-7^(bottom), **e** *P*=9.19x10^-5^*, P*=8.60x10^-5^ (top), *P*=0.0499*, P*=6.47x10^-6^(middle), *P*=0.0152*, P*=8.18x10^-10^(bottom), one-way ANOVA).

**
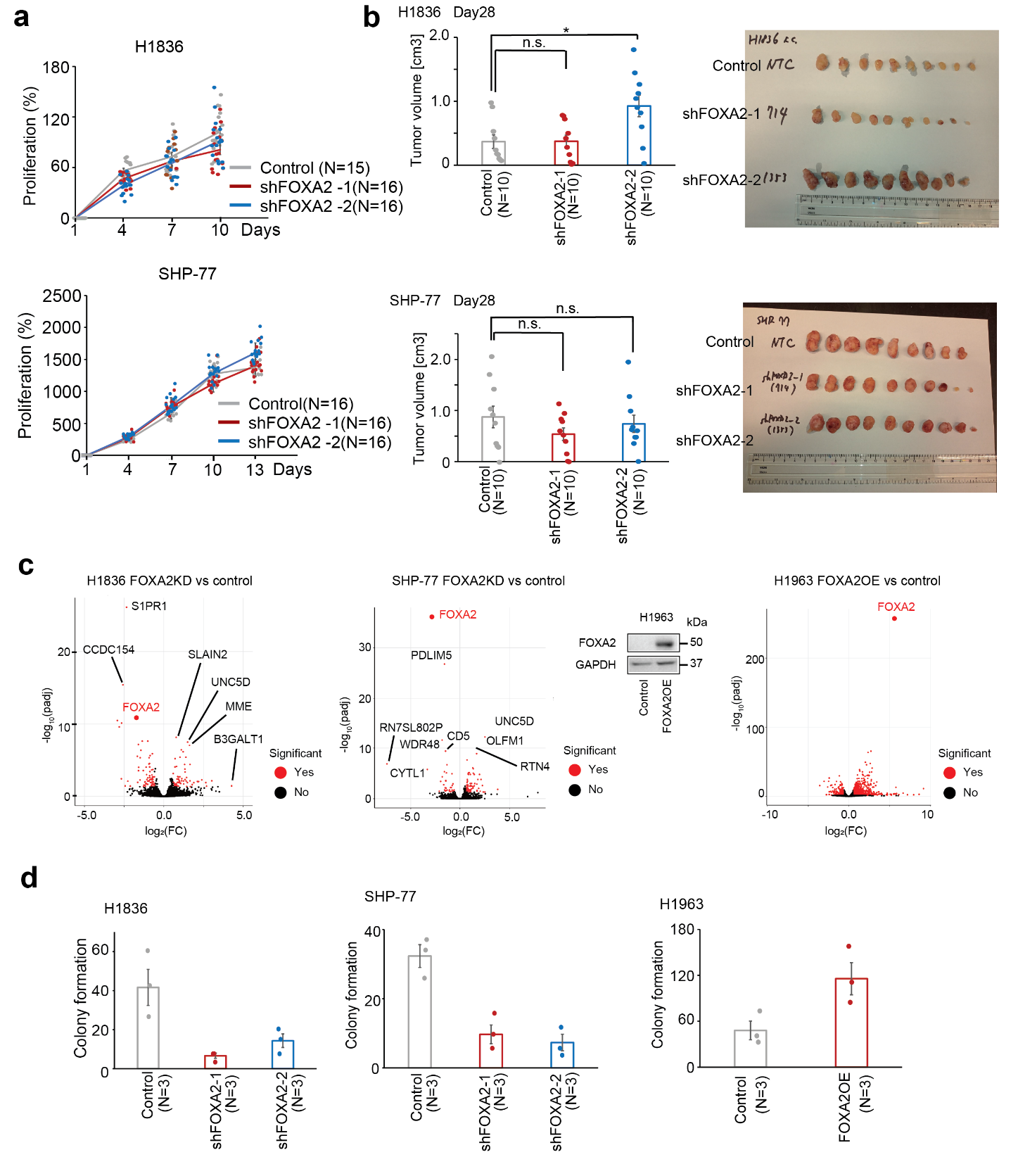
**

**Supplementary Fig 4 related to Figure 4**

**a:** *In vitro* proliferation of FOXA2 KD vs. control cell lines (H1836, SHP-77). Numbers of wells (technical replicates) are indicated as one dot. Mean±SEM. **b:** Tumor volumes at day 28 in control and FOXA2 KD cell lines (H1836, SHP-77). Graphs Mean±SEM (* < 0.05, *P*=0.0117*,* one-way ANOVA). **c:** Volcano plots of gene expression FOXA2 KD vs. control in 2 cell lines (H1836, SHP-77), and FOXA2 OE vs. control (H1963). Western blot of FOXA2 overexpression (OE) is indicated. The samples derive from the same experiment but different gels for FOXA2, another for GAPDH were processed in parallel. A representative data of one of the 3 individual experiments. **d:** Colony formation assay in 2 FOXA2 KD cell lines and FOXA2 OE cell line. Numbers of wells (technical replicates) are indicated as one dot. Graphs Mean±SEM.

**
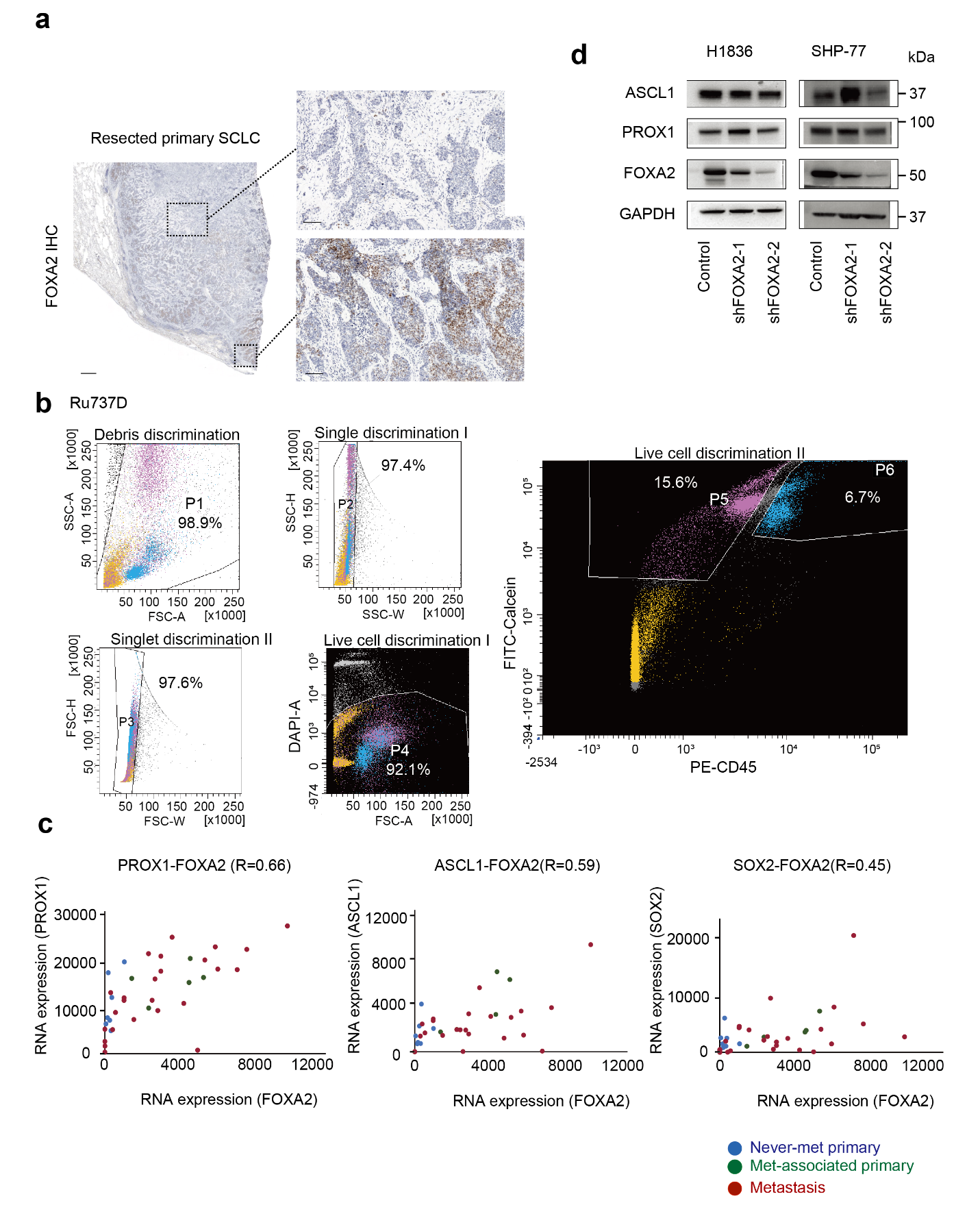
**

**Supplementary Figure 5 related to Figure 5**

**a:** FOXA2 IHC staining of primary SCLC clinical samples indicating FOXA2^-^ and FOXA2^+^ regions. Scale bar: 500μm and 100μm (inset). **b:** FACS gating strategy to generate SCLC scRNA-seq data. The side scatter (SSC-A) and forward scatter (FSC-A) removes debris. Side scatter heights (SSC-H) and side scatter width (SSC-W), forward scatter heights (FSC-H) and forward scatter width (FSC-W) gate single cells. DAPI staining (DAPI negative) selects live-cells. FITC-Calcein and PE-CD45 selects CD45 negative live cells. **c:**Correlation plot of *PROX1* vs. *FOXA2, ASCL1* vs. *FOXA2,* and *SOX2* vs. *FOXA2* expression from the MSKCC SCLC cohort. **d:** Western blot of FOXA2 KD for ASCL1 and PROX1. The samples derive from the same experiment but different gels for ASCL1, FOXA2, another for PROX1 and GAPDH were processed in parallel. A representative data of one of the 3 individual experiments.

**
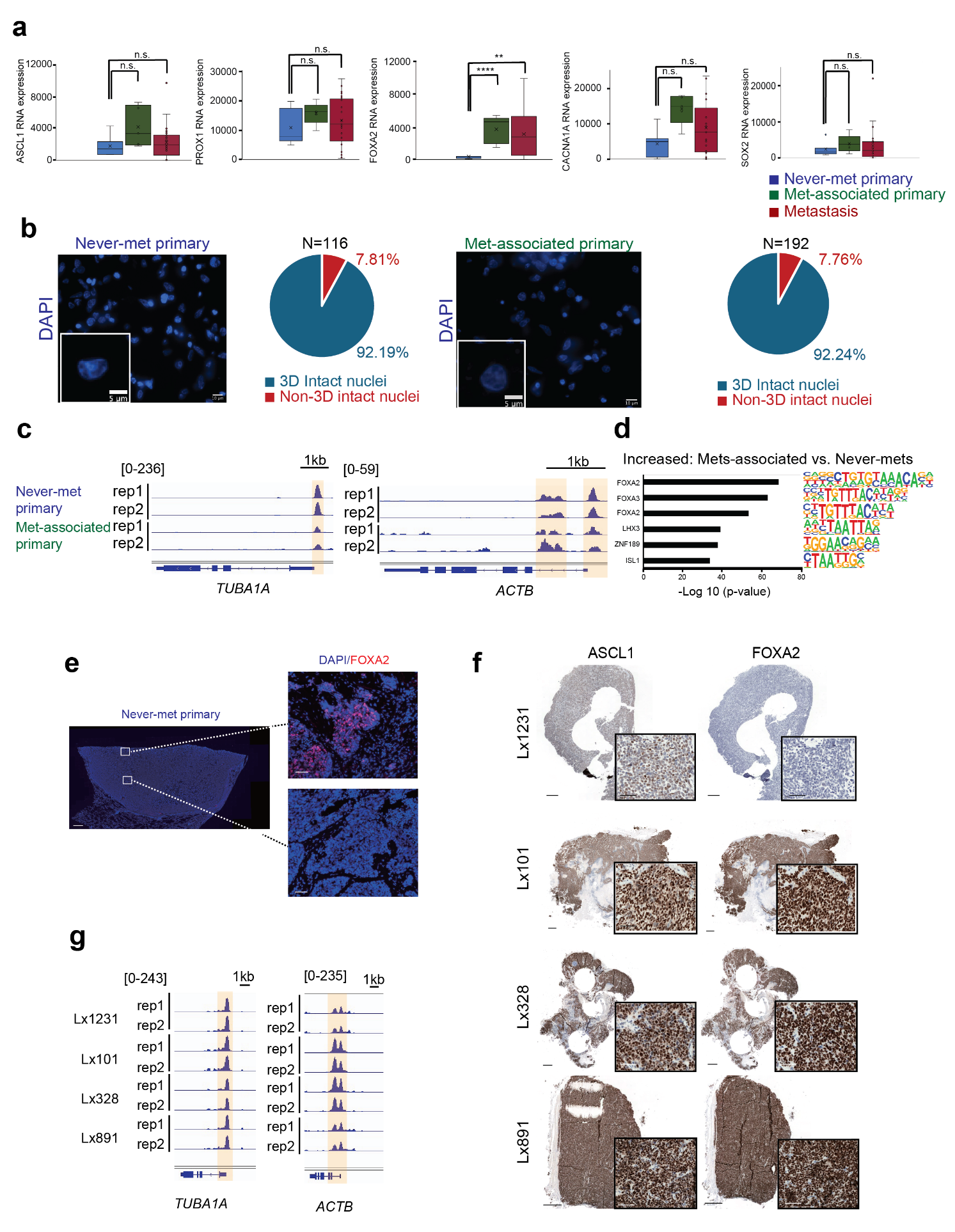
**

**Supplementary Figure 6 related to Figure 6**

**a:** *ASCL1, PROX1, CACNA1A, SOX2* and *FOXA2* expression in never-met primary tumor(N=7), met-associated primary tumor(N=5), and metastases(N=25) in the MSKCC SCLC cohort. Graphs indicate mean±SEM (** < 0.01, **** < 0.0001, FOXA2; *P*=7.31x10^-6^*, P*=8.81x10^-3^, Wald’s t-test). **b:** DAPI staining on never-met primary and met-associated primary FFPE-ATAC processed samples. Scale bar: 10μm (5 μm in inset). **c:** Chromatin accessibility assessed by ATAC-seq at *TUBA1A* and *ACTB* loci (Peaknorm). Highlighted regions indicate promoter domains. **d:** Motif analysis on met-associated primary versus never-met FFPE samples. **e:** Immunofluorescence (IF) image of never-met primary SCLC from Figure 6A for DAPI (blue) and FOXA2 (red). Scale bar: 1000μm (overlay), 50μm (DAPI, FOXA2). **f:** IHC staining of ASCL1^+^FOXA2^+^ PDX tumors (Lx101, Lx328, and Lx891) and ASCL1^+^FOXA2^-^ PDX tumor (Lx1231). Scale bar: 1000μm and 100μm(inset). **g:** ATAC-seq comparing ASCL1^+^FOXA2^+^ PDX (Lx101, Lx328, Lx891) vs ASCL1^+^FOXA2^-^ (Lx1231) PDX tumors at the *TUBA1A and ACTB* loci.
