# Supplementary material for: FOXA2 promotes metastatic competence in small cell lung cancer
Source: Nat Commun. 2025 May 26;16:4865. doi: 10.1038/s41467-025-60141-5 (PMC12106783; doi:10.1038/s41467-025-60141-5)
Supplement: Supplementary file 2 — Description of Additional Supplementary Information [file 41467_2025_60141_MOESM2_ESM.docx]

Supplementary Data

**Table of supplementary content**

-Supplemental table 1: Clinical data of never-metastatic primary cases and metastatic cases

-Supplemental table 2: Gene list of overexpressed and underexpressed genes in metastatic samples

-Supplemental table 3: Clinical data from the samples analyzed for TMA section

-Supplemental table 4: Pathway analysis of RNA-sequence (H1836, SHP-77, H1963)

-Supplemental table 5: Clinical data of SCLC scRNA-seq cases

-Supplemental table 6: Gene list of FOXA2^+^ versus FOXA2^-^ cells on scRNA-seq

-Supplemental table 7: Target sequence of shRNA and PCR primers of ChIP-qPCR in this study
