# Supplementary material for: FOXA2 promotes metastatic competence in small cell lung cancer
Source: Nat Commun. 2025 May 26;16:4865. doi: 10.1038/s41467-025-60141-5 (PMC12106783; doi:10.1038/s41467-025-60141-5)
Supplement: Supplementary file 10 — Reporting Summary [file 41467_2025_60141_MOESM10_ESM.pdf]

nature portfolio

Corresponding author(s): Joan Massagué, Charles M. Rudin

Last updated by author(s): 2025/04/17

Reporting Summary

Nature Portfolio wishes to improve the reproducibility of the work that we publish. This form provides structure for consistency and transparency in reporting. For further information on Nature Portfolio policies, see our Editorial Policies and the Editorial Policy Checklist.

Statistics

For all statistical analyses, confirm that the following items are present in the figure legend, table legend, main text, or Methods section.

n/a	Confirmed	
☐	☒	The exact sample size (n) for each experimental group/condition, given as a discrete number and unit of measurement
☐	☒	A statement on whether measurements were taken from distinct samples or whether the same sample was measured repeatedly
☐	☒	The statistical test(s) used AND whether they are one- or two-sided<br>*Only common tests should be described solely by name; describe more complex techniques in the Methods section.*
☒	☐	A description of all covariates tested
☐	☒	A description of any assumptions or corrections, such as tests of normality and adjustment for multiple comparisons
☐	☒	A full description of the statistical parameters including central tendency (e.g. means) or other basic estimates (e.g. regression coefficient) AND variation (e.g. standard deviation) or associated estimates of uncertainty (e.g. confidence intervals)
☐	☒	For null hypothesis testing, the test statistic (e.g. F, t, r) with confidence intervals, effect sizes, degrees of freedom and P value noted<br>*Give P values as exact values whenever suitable.*
☒	☐	For Bayesian analysis, information on the choice of priors and Markov chain Monte Carlo settings
☒	☐	For hierarchical and complex designs, identification of the appropriate level for tests and full reporting of outcomes
☒	☐	Estimates of effect sizes (e.g. Cohen's d, Pearson's r), indicating how they were calculated

Our web collection on statistics for biologists contains articles on many of the points above.

Software and code

Policy information about availability of computer code

Data collection	In vivo data were collected using Living Image 4.2 (Perkin Elmer). IF image were obtained using Panoramic Scanner (3DHistech).
Data analysis	Software used is as follows;<br>Living Image 4.2 (Perkin Elmer)<br>STAR<br>genecode (v28)<br>DESeq2<br>Gene set enrichment analysis (GSEA, v4.0.2)<br>ClusterProfiler R package v3.18.1<br>Molecular Signatures Database (MSigDB v7.0.1)<br>Panoramic Scanner (3DHistech)<br>Picard Tools v2.16.0<br>TrimGalore<br>FastQC<br>cutadapt<br>MACS2<br>bowtie2<br>Homer v4.5<br>featureCounts

InferCNV (v1.14)
MACS2
featureCounts v1.6.0
BEDTools suite
Integrative Genomics Viewer (IGV)
Scanpy (v1.10)
10x Cell Ranger software (v6.0.1)
Harmonypy (v0.0.9)
Seurat (v5.0)
Biorender Graphic design

For manuscripts utilizing custom algorithms or software that are central to the research but not yet described in published literature, software must be made available to editors and reviewers. We strongly encourage code deposition in a community repository (e.g. GitHub). See the Nature Portfolio guidelines for submitting code & software for further information.

Data

Policy information about availability of data

All manuscripts must include a data availability statement. This statement should provide the following information, where applicable:

- Accession codes, unique identifiers, or web links for publicly available datasets
- A description of any restrictions on data availability
- For clinical datasets or third party data, please ensure that the statement adheres to our policy

Data Availability: Raw sequencing reads and processed files for RNA-seq, ChiP-seq, ATAC-seq and scRNA-seq are deposited in Gene Expression Omnibus database (GEO) under the accession number GSE281523[https://www.ncbi.nlm.nih.gov/geo/query/acc.cgi?acc=GSE281523],GSE281524[https://www.ncbi.nlm.nih.gov/geo/query/acc.cgi?acc=GSE281524],GSE281525[https://www.ncbi.nlm.nih.gov/geo/query/acc.cgi?acc=GSE281525], GSE281740[https://www.ncbi.nlm.nih.gov/geo/query/acc.cgi?acc=GSE281740]. Source Data are provided with this paper. Further information and requests for resources and reagents should be directed to and will be fulfilled by the Lead Contact.

Code Availability: CODE is uploaded to github [https://github.com/shahcompbio/SCLC_MET.git] [DOI: 10.5281/zenodo.15257973].

Research involving human participants, their data, or biological material

Policy information about studies with human participants or human data. See also policy information about sex, gender (identity/presentation), and sexual orientation and race, ethnicity and racism.

Reporting on sex and gender	Sex and gender were not considered to affect the results. As a part of the clinical data, sex and gender are provided in the supplementary table.
Reporting on race, ethnicity, or other socially relevant groupings	Social confounding factors are not considered for our study.
Population characteristics	Population characteristics are provided as the clinical information in the supplementary table 1,3 and 5.
Recruitment	SCLC patients used for this study were enrolled whose tumors were sequenced using the MSK-IMPACT clinical targeted sequencing assay (N=327)
Ethics oversight	The study protocol is approved by Institutional Review Board in Memorial Sloan Kettering Cancer Center

Note that full information on the approval of the study protocol must also be provided in the manuscript.

Field-specific reporting

Please select the one below that is the best fit for your research. If you are not sure, read the appropriate sections before making your selection.

☒ Life sciences ☐ Behavioural & social sciences ☐ Ecological, evolutionary & environmental sciences

For a reference copy of the document with all sections, see nature.com/documents/nr-reporting-summary-flat.pdf

Life sciences study design

All studies must disclose on these points even when the disclosure is negative.

Sample size	No statistical methods were used to pre-determine the sample size used for clinical data collection. We collected the patient data available who has gone through MSK-IMPACT study.<br><br>For all the animal and cell line experiments, at least three replicates or animals per group were used to determine statistical significance. However, no statistical method was used to precisely calculate the sample size.<br>Cell-based experiments were conducted in independent experiments, with cells infected and seeded into technical replicates.<br>Animal studies were conducted using more than three mice per experimental cohort for each genotype (numbers are indicated in each study).

Data exclusions	Clinical samples which has no sufficient materials for DNA/RNA extraction are excluded.
Replication	All in vitro experiments were repeated a minimum of twice independently with similar results (western blots for 3 times (Fig 3k, 5h and 5k)). For FOXA2 KD in cell experiments, two cell lines and two shRNAs targeting distinct sequences of the gene of interest were used. For mouse tumor analysis, intra-cardiac injection were conducted in 2 shRNA, 2 cell lines and more than 2 times showing consistent results. For epigenetic analysis, IgG control and ASCL1 ChiP-seq, and ATAC-seq were performed in two replicates. ChiP-qPCR were performed in 3 replicates. Micrographs are confirmed for the following times as an individual experiment; Fig 3i(twice), 5a and 6a (once in IHC and once in IF in 3 independent patient samples). No statistical method was used to predetermine the sample size.
Randomization	NOD.Cg-Prkdc<scid> Il2rg<tm1Wjl>/SzJ (NSG) mice used in study were all randomly allocated under the age of 6-10 weeks.
Blinding	Data collection, such as tumor measurement, IVIS signals measurement were mostly performed by blinded researchers. For clinical data analysis, such as generating ROC curve and IHC H-score were also performed by independent blinded researcher.

Reporting for specific materials, systems and methods

We require information from authors about some types of materials, experimental systems and methods used in many studies. Here, indicate whether each material, system or method listed is relevant to your study. If you are not sure if a list item applies to your research, read the appropriate section before selecting a response.

Materials & experimental systems

n/a	Involved in the study
☐	☒ Antibodies
☐	☒ Eukaryotic cell lines
☒	☐ Palaeontology and archaeology
☐	☒ Animals and other organisms
☐	☒ Clinical data
☒	☐ Dual use research of concern
☒	☐ Plants

Methods

n/a	Involved in the study
☐	☒ ChIP-seq
☐	☒ Flow cytometry
☒	☐ MRI-based neuroimaging

Antibodies

Antibodies used	ASCL1 (Invitrogen, Cat #:24B72D11, 1:100)<br>ASCL1 (Cell Signaling Technology, Cat #:10585S)<br>ASCL1(Cell Signaling Technology, Cat #: 43666S)<br>Mash1 (Abcam, Cat#:ab211327, 1;1700)<br>NEUROD1 (Abcam, Cat #:ab205300, 1:100)<br>POU2F3 (Santa Cruz, Cat #: sc-293402, 1:1500)<br>Ki67 (Abcam, Cat #: ab16667, 1:1000)<br>PROX1 (Cell Signaling Technology, Cat #:14963S)<br>GAPDH (Cell Signaling Technology, Cat #: 97166S)<br>IgG (Cell Signaling Technology, Cat #: 2729S)<br>FOXA2 (Abcam, Cat #: ab108422, IHC; 1:1000, IF; 1:200)<br>PE anti-CD45 antibody (Biolegend, Cat#: 368510)<br>Calcein AM (Biolegend Cat#: 425201)
Validation	Links to the manufactuer's website;<br>ASCL1 (Invitrogen, Cat #:24B72D11)<br>https://www.thermofisher.com/antibody/product/MASH1-Antibody-clone-24B72D11-Monoclonal/14-5794-82<br>ASCL1 (Cell Signaling Technology, Cat #:10585S)<br>https://www.cellsignal.com/products/primary-antibodies/ascl1-e5s4q-xp-rabbit-mab/10585<br>ASCL1 (Cell Signaling Technology, Cat #: 43666S)<br>https://www.cellsignal.com/products/primary-antibodies/ascl1-e7n9c-rabbit-mab/43666<br>Mash1 (Abcam, Cat#:ab211327)<br>https://www.abcam.co.jp/products/primary-antibodies/mash1achaete-scute-homolog-1-antibody-epr19840-ab211327.html<br>NEUROD1 (Abcam, Cat #:ab205300)<br>https://www.abcam.co.jp/products/primary-antibodies/neurod1-antibody-epr17084-ab205300.html<br>POU2F3 (Santa Cruz, Cat #: sc-293402)<br>https://www.scbt.com/ja/p/pou2f3-antibody-6d1<br>Ki67 (Abcam, Cat #: ab16667)<br>https://www.abcam.co.jp/products/primary-antibodies/ki67-antibody-sp6-ab16667.html<br>PROX1 (Cell Signaling Technology, Cat #:14963S)<br>https://www.cellsignal.com/products/primary-antibodies/prox1-d2j6j-rabbit-mab/14963<br>GAPDH (Cell Signaling Technology, Cat #: 97166S)<br>https://www.cellsignal.com/products/primary-antibodies/gapdh-d4c6r-mouse-mab/97166

IgG (Cell Signaling Technology, Cat #: 2729S)
https://www.cellsignal.com/products/primary-antibodies/normal-rabbit-igg/2729
FOXA2 (Abcam, Cat #: ab108422)
https://www.abcam.co.jp/products/primary-antibodies/foxa2-antibody-epr4466-ab108422.html

Eukaryotic cell lines

Policy information about cell lines and Sex and Gender in Research

Cell line source(s)	H1836 (ATCC, Cat #:CRL-5898), SHP-77 (ATCC, Cat #:CRL-2195), H82 (ATCC, Cat #:HTB-175), and H1963 (ATCC, Cat #:CRL-5982) were purchased from ATCC.
Authentication	Cell lines were authenticated by STR verification
Mycoplasma contamination	All the cell lines were regularly tested for mycoplasma (Universal Mycoplasma Detection Kit, ATCC).
Commonly misidentified lines (See ICLAC register)	This study did not use any cell lines listed as commonly misidentified.

Animals and other research organisms

Policy information about studies involving animals; ARRIVE guidelines recommended for reporting animal research, and Sex and Gender in Research

Laboratory animals	NOD.Cg-Prkdc<scid> Il2rg<tm1Wjl>/SzJ (NSG) mice were used with aged matched between 6 to 10 weeks. Mice were consistently housed and controlled under the environmental conditions of : 21±1.5°C temperature, 55±10% humidity and a 12h light–dark cycle (lights were on from 6:00 to 18:00).
Wild animals	Wild animals were not included in this study.
Reporting on sex	Female mice were used for this study.
Field-collected samples	Field-collected samples were not used in this study.
Ethics oversight	All mice were used and procedures were performed under an approved Institutional Animal Care and Use Committee protocol from MSKCC

Note that full information on the approval of the study protocol must also be provided in the manuscript.

Clinical data

Policy information about clinical studies
All manuscripts should comply with the ICMJEguidelines for publication of clinical research and a completedCONSORT checklist must be included with all submissions.

Clinical trial registration	Clinical trial registration is not performed since this is a retrospective data analysis.
Study protocol	Study protocol of the definition of "never-metastatic SCLC" and "metastasis-associated" ("met-associated") primary SCLC is written in the manuscript. Never-metastatic criteria included pathologic stage T1-3N0M0, definitive treatment with surgical resection or concomitant chemoradiation, no relapse within a minimum of 2 years of documented follow-up, and available tumor material.
Data collection	The clinical cohort described here are patients with SCLC whose tumors were sequenced using the MSK-IMPACT clinical targeted sequencing assay (N=327) and were analyzed under an Institutional Review Board approved protocol.
Outcomes	This is a retrospective data analysis and clinical outcomes were not measured.

Plants

Seed stocks	Seeds were not used in this study.
Novel plant genotypes	Plants were not used in this study.
Authentication	N/A

ChIP-seq

Data deposition

- [x] Confirm that both raw and final processed data have been deposited in a public database such as GEO.
- [x] Confirm that you have deposited or provided access to graph files (e.g. BED files) for the called peaks.

Data access links<br>*May remain private before publication.*	We deposited these data and we added the GEO accession numbers to the manuscript;<br>GSE281523, GSE281524, GSE281525, GSE281740<br>CODE is uploaded to Github; https://github.com/shahcompbio/SCLC_MET.git
Files in database submission	*Provide a list of all files available in the database submission.*
Genome browser session<br>(e.g. UCSC)	*Provide a link to an anonymized genome browser session for "Initial submission" and "Revised version" documents only, to enable peer review. Write "no longer applicable" for "Final submission" documents.*

Methodology

Replicates	2 replicates were generated for IgG control and ASCL1 ChiP-seq.
Sequencing depth	An average of 20-30 million paired reads were generated per sample.
Antibodies	IgG: Cell Signaling Technology, Cat #: 2729S<br>ASCL1: Cell Signaling Technology, Cat #: 43666S
Peak calling parameters	Enriched binding regions were called against the input or IgG reference samples using MACS2 with p value<0.001
Data quality	Enriched binding regions were called against the input or IgG reference samples using MACS2 with p value<0.001
Software	The software used for the process are<br>MACS2<br>featureCounts v1.6.0<br>DESeq2<br>BEDTools suite<br>Integrative Genomics Viewer (IGV)

Flow Cytometry

Plots

Confirm that:

- [x] The axis labels state the marker and fluorochrome used (e.g. CD4-FITC).
- [x] The axis scales are clearly visible. Include numbers along axes only for bottom left plot of group (a 'group' is an analysis of identical markers).
- [x] All plots are contour plots with outliers or pseudocolor plots.
- [x] A numerical value for number of cells or percentage (with statistics) is provided.

Methodology

Sample preparation	Clinical samples were treated in the same way as [Chan J et al. Cancer Cell, 2021, Quintanal-Villalonga, Á. et al. STAR Protoc, 2022]. In brief, the cells are stained for sorting and CD45+ composition analyses Cell pellet was resuspended in 200-3000 uL of Red Blood Cell Lysis Solution (ACK lysis buffer). Cell pellet was resuspended in 100 uL of 1xPBS+2.5%FBS, mixedwith5uL of HumanTruStainFcX (Biolegend#422302), 3 uL of CD45antibody(Biolegend #368510 and 0.1uL of calcein (1mg/mL, Calcein (Biolegend #425201)), and left for 15 minutes on ice. Stained samples were washed twice with 2 mlof1xPBS+2.5%FBS,and finally resuspended in the same buffer supplemented with DAPI dye.
Instrument	BD FACS Aria (BD Biosciences) or Sony MA900 (Sony) flow cytometers
Software	N/A
Cell population abundance	CD45- in the live cell (DAPI-, Calcein+) population
Gating strategy	Cells were sorted on DAPI-, Calcein+ (FITC+) to select for live cells. In addition, we sorted CD45+ (immune cells) and CD45- (cell population enriched in cancer cells) populations into separate tubes.Please refer to FigS5B.

- [x] Tick this box to confirm that a figure exemplifying the gating strategy is provided in the Supplementary Information.
